# P-1767. A Gut Microbiome Specific Antibiotic Spectrum Index (gASI) Predicts Clostridioides difficile Infection Disease Severity

**DOI:** 10.1093/ofid/ofaf695.1937

**Published:** 2026-01-11

**Authors:** Taryn A Eubank, Abdulwhab A Shremo Msdi, Kevin W Garey

**Affiliations:** University of Houston College of Pharmacy, Houston, TX; University of Houston College of Pharmacy, Houston, TX; University of Houston College of Pharmacy, Houston, TX

## Abstract

**Background:**

Antibiotics are the top modifiable risk factor for *Clostridioides difficile* infection (CDI) thus is a recognized antimicrobial stewardship initiative. Antibiotic spectrum index (ASI) has been developed to better quantify antibiotic impact and stewardship practices. Recently, ASI score was associated with hospital-acquired CDI. This study aims to investigate ASI score correlation with CDI disease severity and pilot a gut microbiome specific ASI (gASI).

**Figure 1.**
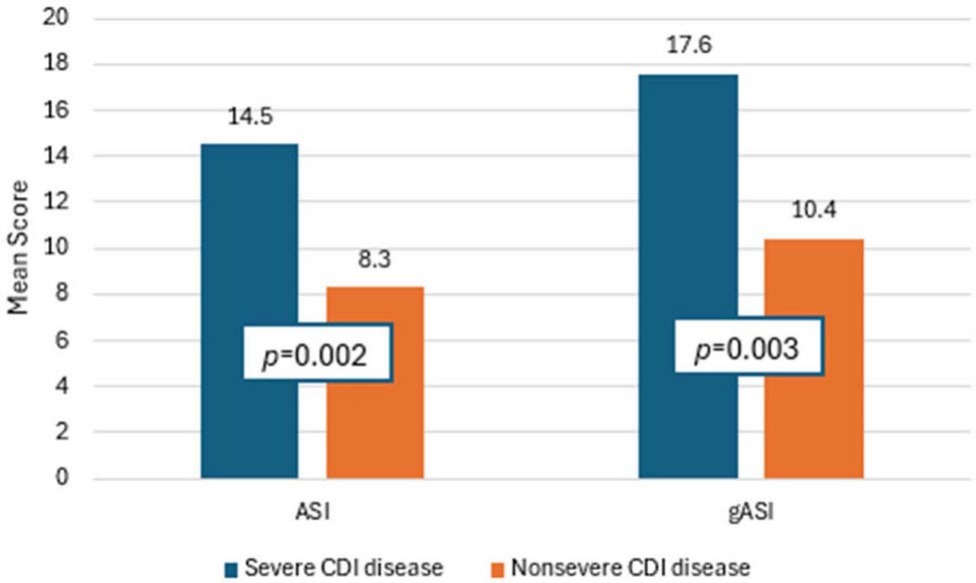
Comparison of mean ASI scores and gASI scores associations with CDI disease severity

**Table 1.**
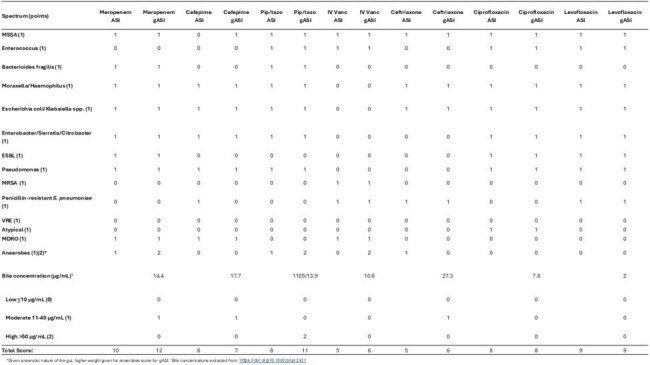
Score differences between ASI and gASI of top antibiotic exposures.

**Methods:**

We performed a case-control study of adult patients hospitalized with CDI from two health systems (14 hospitals) in Houston, TX USA (2016-2024). Patients were selected based on CDI disease severity and matched on variables known to impact severity including immunocompromised status and age ±10 years. Additional clinical variables of interest were extracted from the electronic health record with detailed documentation of duration and type of antibiotic exposure in the previous 30 days prior to CDI diagnosis. Disease severity and CDI classification were defined according to the 2017 IDSDA/SHEA clinical guidelines. gASI development utilized previously published ASI score with additional points for anaerobic coverage to emphasize microbiota impact and antibiotic present in bile as a surrogate for microbiota exposure.

**Results:**

A total of 100 patients (50 severe vs 50 nonsevere) with CDI were included (Female: 53%, white, non-Hispanic: 54%; Age > 65 years: 66%; hospital-acquired CDI: 50%; CDI initial episode: 89%). Higher ASI score was significantly associated with severe CDI (14.5±10.9 vs 8.3±8; *p*=0.002) (Figure 1). Table 1 demonstrates the score differences between ASI and gASI. gASI remained significantly associated with severe CDI (17.6±13.3 vs 10.4±10; *p*=0.003). Patients with severe CDI had significantly more exposure to intravenous vancomycin, cefepime, piperacillin/tazobactam, and meropenem.

**Conclusion:**

ASI and newly developed gASI both correlate with CDI disease severity. Future work to validate and strengthen gASI score is warranted through metagenomics and/or metabolomics to capture the true impact of antibiotics on the gut microbiome.

**Disclosures:**

Kevin W. Garey, MS;PharmD, Acurx: Grant/Research Support|Merck: Grant/Research Support|Paratek: Grant/Research Support

